# Exploring adsorption capacity and mechanisms involved in cadmium removal from aqueous solutions by biochar derived from euhalophyte

**DOI:** 10.1038/s41598-023-50525-2

**Published:** 2024-01-03

**Authors:** Shaoqing Ge, Shuai Zhao, Lei Wang, Zhenyong Zhao, Shoule Wang, Changyan Tian

**Affiliations:** 1 State Key Laboratory of Desert and Oasis Ecology, Xinjiang Institute of Ecology and Geography, Chinese Academy of Sciences, 818 South Beijing Road, Ürümqi, 830011 Xinjiang China; 2 Shandong Institute of Pomology, Taian, 271000 China

**Keywords:** Ecology, Environmental sciences

## Abstract

Biochar has shown potential as a sorbent for reducing Cd levels in water. Euhalophytes, which thrive in saline-alkali soils containing high concentrations of metal ions and anions, present an intriguing opportunity for producing biochar with inherent metal adsorption properties. This study focused on biochar derived from the euhalophyte *Salicornia europaea* and aimed to investigate its Cd adsorption capacity through adsorption kinetics and isotherm experiments. The results demonstrated that *S. europaea* biochar exhibited a high specific surface area, substantial base cation content, and a low negative surface charge, making it a highly effective adsorbent for Cd. The adsorption data fit well with the Langmuir isotherm model, revealing a maximum adsorption capacity of 108.54 mg g^−1^ at 25 °C. The adsorption process involved both surface adsorption and intraparticle diffusion. The Cd adsorption mechanism on the biochar encompassed precipitation, ion exchange, functional group complexation, and cation-π interactions. Notably, the precipitation of Cd^2+^ with CO_3_^2−^ in the biochar played a dominant role, accounting for 73.7% of the overall removal mechanism. These findings underscore the potential of euhalophytes such as *S. europaea* as a promising solution for remediating Cd contamination in aquatic environments.

## Introduction

Cadmium (Cd) is a highly prevalent metal pollutant in water, presenting significant risks to both ecological and human health due to its high biological toxicity, mobility, and potential for accumulation^1^. According to the U.S. Environmental Protection Agency (EPA), the maximum allowable level of cadmium in drinking water is 0.005 mg per liter^2^. Various techniques are available for removing Cd from wastewater, including solvent extraction, chemical precipitation, membrane filtration, electrochemical methods and adsorption^3–5^. Considering various techniques, the adsorption of Cd using adsorbents is an economically viable method for remediating contaminated water^6^.

Biochar, derived from biomass (such as plant residues, agricultural waste, and manure) through pyrolysis under oxygen-limited conditions, is a carbon-rich byproduct. It serves as a widely used, cost-effective adsorbent for various pollutants in aqueous solutions^7,8^. The mechanism of of Cd^2+^ adsorption on biochar has been undertaken to studied extensively, uncovering several key processes: (1) Precipitation involving minerals present in biochar, such as CO_3_^2−^, OH^−^, PO_4_^3−^, and SO_4_^2−^^9–11^. (2) Exchange of Cd^2+^ with other metal ions, such as Ca^2+^, Mg^2+^, K^+^, Na^+^, and –COOM^12^. (3) Surface complexation with oxygen-containing functional groups, such as –OH and –COOH^13^. (4) Cation-π interactions involving aromatic systems, C=C, and C=O^14,15^. These investigations have provided valuable insights into the diverse mechanisms underlying Cd^2+^ adsorption onto biochar, highlighting the complex nature of the adsorption process.

The adsorption capacity of biochar for Cd^2+^ has demonstrated limitations in prior tests. For instance, biochars derived from maize stalks (pyrolyzed at 500 °C) and rapeseed straw (pyrolyzed at 600 °C) exhibited Cd^2+^ adsorption capacities of 12.67 mg g^−1^ and 32.74 mg g^−1^, respectively^16,17^. Similarly, livestock manure-derived biochar (pyrolyzed at 450 °C) demonstrated a Cd^2+^ adsorption capacity of 8.89 mg g^−1^^18^. The constrained adsorption capacity of these biochars for heavy metals is frequently attributed to their insufficient mineral content^19^. Consequently, biochar is often subjected to modifications to boost its mineral content, thereby creating more binding sites on its surface and enhancing its adsorption capacity for Cd^20,21^. Notably, recent studies have discovered that biochar pyrolyzed from euhalophytes naturally contains a substantial amount of mineral content and functional group^22,23^, This revelation implies the potential of biochar from euhalophyte as an efficient Cd adsorbent. However, our comprehension of the adsorption capacity of biochar derived from euhalophyte is still in its early stage.

Euhalophytes, which can complete their life cycle in saline soils, produce substantial biomass in areas where glycophytes cannot thrive^24^. *Salicornia europaea*, a leaf succulent annual euhalophyte, is widely found in saltmarshes and mudflats worldwide. It exhibits a remarkable ability to accumulate high quantities of ions in its tissues and can yield over 11,000 kg of biomass per hectare^25^. As part of soil salinity reduction practices, *S. europaea* is often harvested at the end of the growing season^26^, potentially leaving behind metal ions (e.g., Na^+^, K^+^, Ca^2+^) and anions (e.g., CO_3_^2−^, OH^−^) in its biomass. We hypothesized that biochar derived from this euhalophyte could exhibit a robust adsorption capacity for cadmium. This is due to these ions facilitating ion exchange or Cd precipitation. Gaining further insight into biochars derived from euhalophytes would not only contribute to the utilization of plant residues but also offer innovative materials for remediating Cd pollution in aquatic environments.

In this study, biochar was generated from the biomass of *Salicornia europaea*. To gain insights into its characteristics, we performed batch adsorption experiments. Our primary objective was to investigate the adsorption behavior of Cd^2+^ ions by the biochar derived from *S. europaea* in aqueous solutions, and subsequently elucidate the underlying adsorption mechanism. To establish a benchmark for comparison, *Zea mays*-derived biochar was employed as a control, as it is widely utilized in the remediation of both aquatic and soil environments.

## Materials and methods

### Biochar prepared

The *S. europaea* sample was obtained from Karamay city (45° 28′ 6.38″ N, 84° 59′ 41.61″ E), Xinjiang Province, China.The samples were taken to the Laboratory of Fundamental Biology, Xinjiang Institute of Ecology and Geography to be labeled as specimen number 102, and was identified by Dr. Zumei Mao as *Salicornia europaea*. Sample collection has complied with relevant institutional, national, and international guidelines and legislation. The preparation and basic physicochemical properties of *S. europaea*-derived biochar have been mentioned in Ge et al.^22^. The photos of raw and biochar of *S. europaea* are presented in Fig. S1a, b, respectively. The *Z. mays*-derived biochar was purchased from Institute of Soil Science, Chinese Academy of Sciences, Nanjing, China. The biochar derived from *Salicornia europaea* and *Zea mays* were denoted as SBC (*Salicornia europaea-* biochar) and ZBC (*Zea mays*-biochar), respectively.

To remove minerals present in the biochars, as described by Qiu et al.^27^ demineralization was carried out by rinsing the biochars with 1 M HCl solution. This was followed by washing with distilled water several times until the pH of the rinsing solution reached a constant value.The demineralized SBC and ZBC were referred to as SBCA and ZBCA, respectively, for further analysis.

### Trait analyse

The surface morphology and element distributions of the biochars were analyzed using scanning electron microscopy–energy dispersive X-ray spectroscopy (SEM–EDS) with a Zeiss Supra 55VP instrument (Oregon, USA). The pore structure of the biochars was assessed by N_2_ adsorption at 77 K using a Nova 2200e surface area analyzer (Quantachrome Instruments, Florida, USA). The specific surface area (SSA) was calculated using the BET (Brunauer–Emmett–Teller) method, while the average pore volume was determined using the BJH (Barrett-Joyner-Halenda) method.

Zeta potential measurements were performed by dispersing the biochar in solutions ranging from pH 2.0 to 9.0, and the measurements were conducted using a Zetasizer instrument (Nano-ZS90, Malvern, UK). X-ray diffraction was performed using a computer-controlled diffractometer (D8 Advance, Bruker, Germany) with Al-Kα radiation (K-Alpha, Thermo Scientific, USA). Fourier transform infrared spectroscopy (FTIR) was conducted using a Nicolet 6700 instrument (USA) in the wavelength range of 400–4000 cm^−1^. X-ray photoelectron spectra were acquired using Al-Kα X-rays and a K-Alpha instrument (Thermo Fisher Scientific).

### Adsorption capacity measurement

The impact of pH on Cd (II) adsorption by the biochar was investigated within an initial pH range of 2–9. Each adsorbate solution (30 mL) with an initial Cd^2+^ concentration of 30 mg L^−1^ was added to 50 mL polypropylene centrifuge tubes. Subsequently, 20 mg of biochar was introduced to each vial, and the mixtures were allowed to react for 8 h at a temperature of 25 °C ± 0.5 °C.

For the adsorption isothermal experiments, a series of Cd(NO_3_)_2_ solutions ranging from 5 to 100 mg L^−1^ were prepared. The initial pH of the cadmium solution was adjusted to 6.0 ± 0.1 using 0.01 M HNO_3_ or NaOH solution to prevent Cd precipitation. Then, 20 mg of biochar and 30 mL of Cd(NO_3_)_2_ solution (5–100 mg L^−1^) were combined in 50 mL polypropylene centrifuge tubes. The mixtures were shaken at 150 rpm for 8 h at 15, 25 and 35 °C ± 0.5 °C, respectively, followed by centrifugation at 4000 rpm for 10 min. The supernatants were filtered through 0.45 μm filter papers for subsequent Cd concentration analysis.

Regarding the adsorption kinetics experiments, 20 mg of biochar and 30 mL of Cd(NO_3_)_2_ solution (30 mg L^−1^) were mixed in 50 mL polypropylene centrifuge tubes under the same conditions as the isothermal experiment. The mixtures were treated similarly, and adsorption equilibrium was achieved after shaking. The reaction time was measured at 1, 2, 3, 4, 5, 6, 7, 8, 9, 10, 20, 30, 40, 50, 60, 80, 100, and 120 min, respectively.

All treatments were conducted in quadruplicate. The biochar samples were dried for SEM–EDS, XRD, FTIR, and XPS analyses. The Cd concentration was determined using inductively coupled plasma optical emission spectroscopy (ICP-OES; Agilent 735, USA).

### Assessment of Cd^2+^ adsorption mechanisms

The contributions of different mechanisms to Cd^2+^ adsorption on the biochars were determined using a modified version of the method proposed by Wang et al.^9^. The adsorption capacities attributed to metal ion exchange (*Q*_*CMe*_), precipitation with minerals (*Q*_*CMp*_), functional group complexation (*Q*_*CO*_), and Cd^2+^–π interactions (*Q*_*Cπ*_) were determined as follows.

1. In this study, almost no cations (K^+^, Na^+^, Ca^2+^, and Mg^2+^) were observed in the solutions of SBCA and ZBCA (Table S1), implying that adsorption by residual minerals was negligible. Hence, the reduction in the amount of Cd^2+^ sorbed on the biochars after demineralization could be considered as the contribution of these removed minerals^27^. The amount of Cd^2+^ adsorption attributed to the interaction with minerals (*Q*_*CM*_, in mg g^−1^) was calculated as
  1 $$Q_{CM} = Q_{CT} - Q_{CA} \times Y$$
  where *Q*_*CT*_ (mg g^−1^) is the total adsorption of Cd^2+^ on SBC or ZBC, *Q*_*CA*_ (mg g^−1^) is the amount of sorbed Cd^2+^ on SBCA or ZBCA, and *Y* is the yield of demineralized biochar from the original biochar.
2. The amount of exchanged base cations (K^+^, Ca^2+^, Na^+^, and Mg^2+^) released from the biochars was determined by the difference in the concentration of these cations in solution before and after Cd^2+^ adsorption. Thus, the amount of Cd^2+^ adsorption resulting from ion exchange (*Q*_*CMe*_) was defined as the sum of exchanged cations:
  2 $$Q_{CMe} = Q_{K} + Q_{Na} + Q_{Ca} + Q_{Mg}$$
  where* Q*_*K*_, *Q*_*Na*_, *Q*_*Ca*_, and *Q*_*Mg*_ are the net amounts of K, Ca, Na, and Mg released from SBC or ZBC into solution after Cd^2+^ adsorption, respectively. The calculation was normalized to mEq L^−1^.
3. The adsorption of Cd^2+^ on minerals resulted from a combination of ion exchange and mineral precipitation. Thus, the amount of Cd^2+^ adsorption resulting from mineral precipitation (*Q*_*CMp*_) could be calculated by the difference between *Q*_*CM*_ and *Q*_*CMe*_:
  3 $$Q_{CMp} = Q_{CM} - Q_{CMe}$$
4. When the biochars were deashed, the decrease in pH upon Cd^2+^ adsorption on SBCA and ZBCA was attributed to coordination with oxygen-containing organic groups, which can be described by the following reactions^13^:
  4 $$- {\text{COOH }} + {\text{ Cd}}^{{{2} + }} + {\text{ H}}_{{2}} {\text{O }} \to - {\text{COOCd}}^{ + } + {\text{ H}}_{{3}} {\text{O}}^{ + }$$
  5 $${-}{\text{OH }} + {\text{ Cd}}^{{{2} + }} + {\text{ H}}_{{2}} {\text{O }} \to \, {-}{\text{OCd}}^{ + } + {\text{ H}}_{{3}} {\text{O}}^{ + }$$

The amount of H^+^ released was calculated from the decrease in pH, and the amount Cd^2+^ adsorption through functional group complexation (*Q*_*CO1*_) was calculated accordingly. The adsorption attributed to complexation with oxygen-containing functional groups (*Q*_*CO*_) was calculated as

6 $$Q_{CO} = Q_{CO1} \times Y$$

(5) Cd^2+^ adsorption on SBCA and ZBCA resulted from a combination of Cd^2+^–π interactions and functional group complexation. Thus, the amount of Cd^2+^ adsorption through Cd^2+^–π interactions (*Q*_*Cπ*_) was calculated by the difference between the *Q*_*CA*_ and *Q*_*CO*_ values of SBCA and ZBCA:

7 $$Q_{C\pi } = Q_{CA} \times Y - Q_{CO}$$

In addition, the percentage contributions of the different mechanisms to Cd^2+^ adsorption were calculated as the *Q*_*CMe*_/*Q*_*CT*_, *Q*_*CMp*_/*Q*_*CT*_, *Q*_*CO*_/*Q*_*CT*_, and *Q*_*Cπ*_/*Q*_*CT*_ ratios.

### Regeneration test

The leaching experiment was carried out to evaluate the stability of biochar for Cd adsorption. The filling height was 30 cm, and inner diameter 5 cm soil column was filled with 10 mg kg^−1^ Cd polluted soil (control group) and 1% SBC, and 2% SBC-amended soil (treated groups), respectively. The following materials were placed in the tube from bottom to top: a layer of filter paper, quartz, a layer of filter paper, 350 g of soil, a layer of filter paper, non-woven fabric. The quartz was washed with 0.01 M NaOH and 0.01 M HCl and dried at 105 °C. Soil density was similar to that of the original soil. Deionized water was poured into the soil columns from the top. Leachate was collected in 50 mL aliquots in a conical flask on 1, 3, 5, 7, 9, 11, 13, 15, 17, 19, 21 days. The leachate was filtered through a 0.45-µm membrane filter for analysis of the heavy metal concentrations.

### Statistical analyses

The statistical analyses were performed using SPSS 19.0 (IBM SPSS Inc., Chicago, IL, United States). One-way analysis of variance (ANOVA) was employed to test for significant differences among the treatments. For multiple comparisons of the mean values, the Tukey test was applied (*P* < 0.05). All the reported results were based on quadruplicate experimental replicates.

## Results and discussion

### Pore structure and surface charge characterization

**Figure 1:**
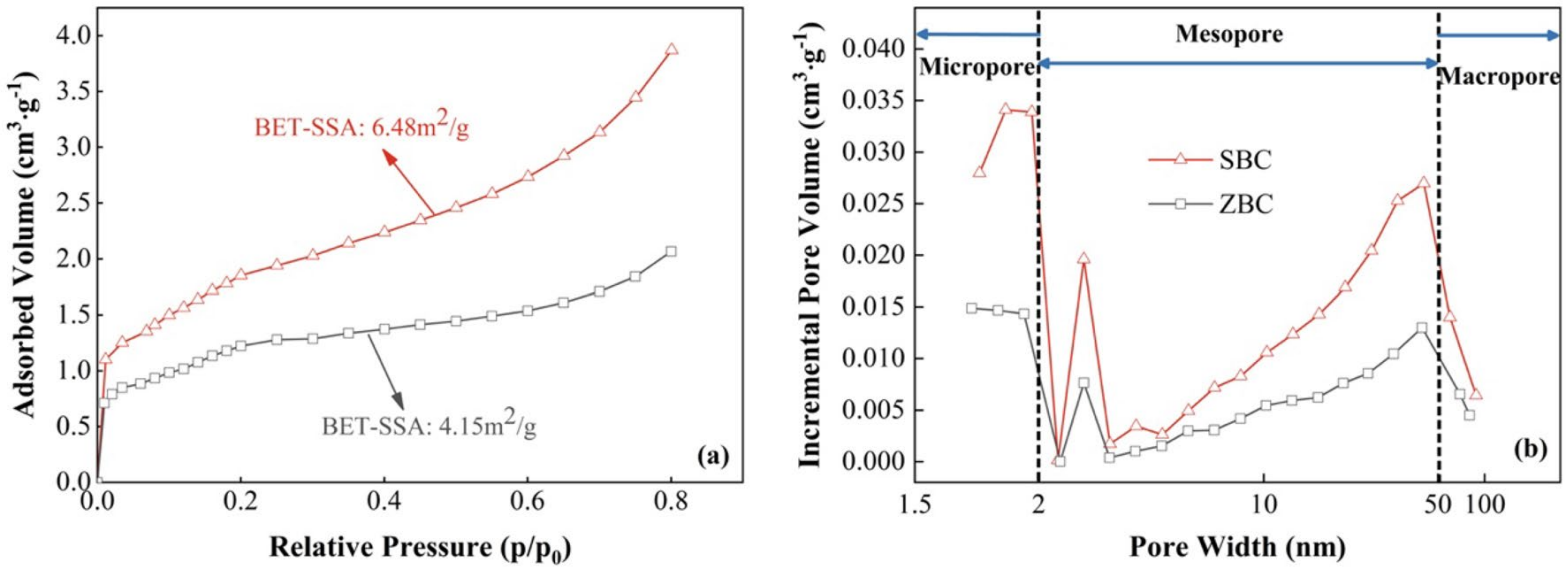
Characterization of the biochar pore structure. (**a**) N_2_ adsorption–desorption isotherms and (**b**) corresponding pore size distributions based on the Barrett–Joyner–Halenda (BJH) method. SBC, *Salicornia europaea*- biochar; ZBC, *Zea mays*-biochar.

The pore structure of biochar plays a significant role in assessing its adsorption efficiency^28^. In this study, the isotherm shapes of the biochars were classified as approximately type IV according to the IUPAC classification system^29^. The SBC exhibited a specific surface area of 6.48 m^2^ g^−1^, which was 56% higher than that of the ZBC (Fig. 1a). This increased specific surface area provides abundant adsorption energy and numerous adsorption sites for Cd^2+^^30^, as the adsorption capacity of biochar is typically proportional to its surface area^31^. Moreover, the SBC displayed a dominant presence of micropores and mesopores, as indicated by the average pore width (4 V A^−1^ based on BET measurements) (Fig. 1b). This pore structure is advantageous for the adsorption of heavy metal cations^32^.

The Zeta points of the two biochars under different pH conditions are presented in Fig. S2. As observed, the Zeta point of the biochar decreased with increasing pH value. The surface of the SBC exhibited a negative charge at pH > 2. Compared to the ZBC, the SBC had a smaller zero charge. When the pH of the medium exceeds the point of zero charge (PZC) of the biochar, electrostatic adsorption occurs, allowing for the adsorption of positively charged ions^33^.

### Solution pH dependent Cd^2+^ adsorption efficiency

**Figure 2:**
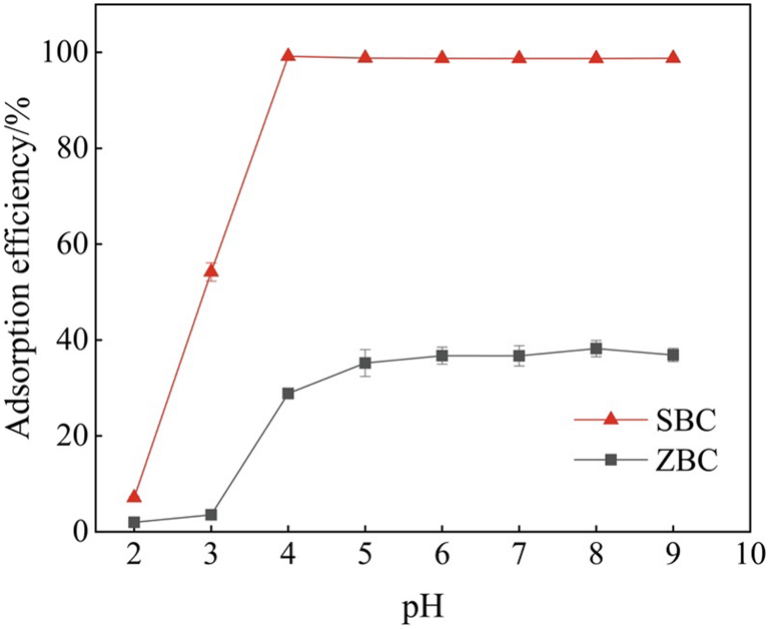
Investigation of pH influence on Cd (II) adsorption by *S. europaea*-biochar (SBC) (**a**) and *Z. mays*-biochar (ZBC) (biochar dosage 20 mg, initial Cd^2+^ concentrations 30 mg L^−1^, contact time 8 h, temperature 25 °C).

The pH value of the solution is a critical parameter that significantly affects the metal adsorption process^34^. In this study, we examined the effect of pH on the removal of Cd by SBC by varying the pH of the solution within the range of 2.0–9.0. As illustrated in Fig. 2, the adsorption efficiency of Cd^2+^ by different biochars increased with the rise in initial solution pH. In the pH range of 2.0–4.0, the adsorption of Cd on SBC exhibited a rapid increase, reaching a maximum adsorption efficiency of 99.2% at pH 4 and then remained stable. Conversely, ZBC exhibited the highest adsorption capacity at pH 5.0.

The influence of pH on Cd^2+^ adsorption can be attributed to the surface charge of the adsorbent. When the pH of the solution is below 3.0, the surface of SBC carries a positive charge. This positive charge leads to repulsion, limiting the proximity of Cd^2+^ ions and promoting competition between H^+^ and Cd^2+^ for active sites on SBC. Conversely, when the pH exceeds 3.0, the surface of SBC becomes negatively charged, while Cd^2+^ ions are positively charged. As a result, the adsorption is driven by the electrostatic interaction between Cd^2+^ and SBC^35^. Furthermore, as the pH increases, the competition of H^+^ for active sites weakens, which explains the enhanced adsorption capacity with higher pH values. Remarkably, even at pH > 7, where the competition of H^+^ becomes negligible, SBC still exhibits a strong adsorption capacity, potentially attributed to the precipitation of Cd^36^.

### The isotherms involved in the Cd^2+^ adsorption

**Figure 3:**
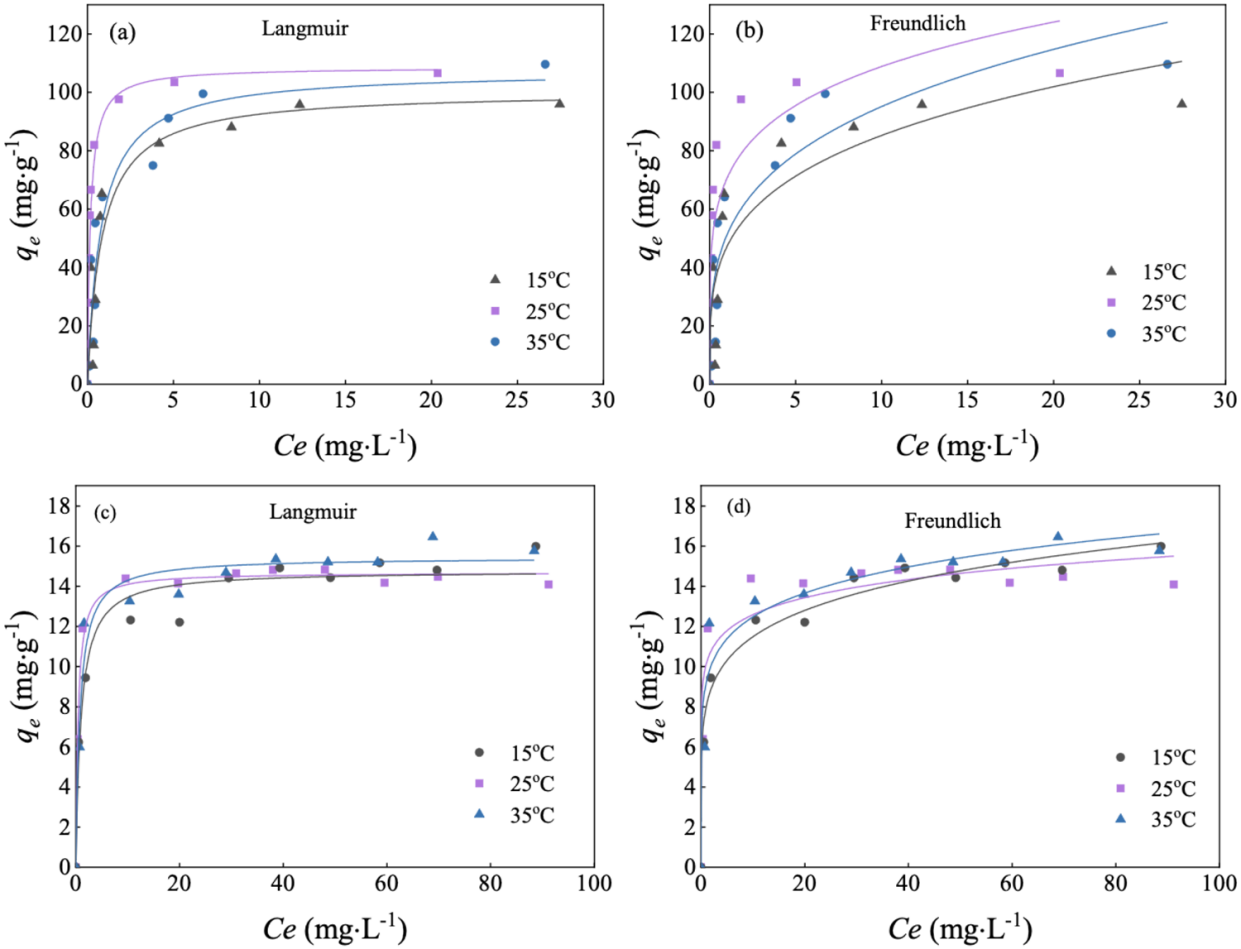
Isothermal adsorption test for Cd^2+^ on *S. europaea*-biochar (SBC) (**a**) and *Z. mays-* biochar (ZBC) (**b**). (Biochar dosage 20 mg, initial Cd^2+^ concentrations ranging from 5 to 100 mg L^−1^, contact time 8 h, pH 6.0).

The adsorption capacity of SBC for Cd^2+^ was significantly higher compared to ZBC (Fig. 3). The Langmuir model provided the best fit for the isothermal adsorption curves of SBC (Table 1), indicating that Cd^2+^ adsorption primarily occurred in monolayers with homogeneous active sites on the biochar^37^. The 1/n values ranging from 0.1 to 0.5 for SBC suggested that the main adsorption process took place on its surface^38^. SBC exhibited a maximum adsorption capacity of 108.54 mg g^−1^, which was 7.4 times higher than that of ZBC (14.69 mg g^−1^) at 25 °C. The higher ash content in SBC was found to promote the adsorption of Cd^39^, which may explain its higher adsorption capacity. According to the Langmuir model, the adsorption isotherms of both SBC and ZBC reached a plateau at high Cd concentrations, indicating adsorption saturation.

**Table 1:** Fitting parameters for the Cd adsorption isotherms of *S. europaea* biochar (SBC) and *Z. mays* biochar (ZBC) (biochar dosage 20 mg, initial concentrations of Cd^2+^ 5–100 mg L^−1^, contact time 8 h, pH 6.0).

Adsorbent	^o^C	Langmuir parameters			Freundlich parameters			ACPA (mg m^−2^)
		*b* (L mg^−1^)	*q*_m_ (mg g^−1^)	R^2^	*K*_*f*_ (mg^1−1/n^ g^−1^ L^1/n^)	1/*n*	R^2^	
SBC	15	1.18	100.28	0.89	47.04	0.26	0.80	16.75
	25	6.04	108.54	0.97	70.42	0.19	0.81	
	35	1.24	107.33	0.90	51.03	0.27	0.84	
ZBC	15	1.01	14.18	0.98	8.01	0.15	0.96	3.54
	25	2.37	14.69	0.99	10.13	0.09	0.92	
	35	1.25	15.44	0.96	9.21	0.13	0.94	

According to the Langmuir model, both SBC and ZBC exhibited adsorption isotherms reached a plateau at high Cd concentrations, indicating saturation of adsorption. Additionally, the results of thermodynamic parameters showed that the ΔH^0^ of SBC was negative, suggesting that the adsorption of Cd^2+^ on SBC is an exothermic process. This could explain the observed lower adsorption capacity of Cd^2+^ by SBC at 35 °C compared to 25 °C (Table 1). In contrast, the ΔH^0^ of ZBC was positive, and the adsorption capacity increased with temperature, signifying that the adsorption of Cd^2+^ on ZBC is an endothermic process^40^.

The decrease in the *R*_*L*_ value with increasing Cd concentration suggested that higher initial Cd concentrations favored biochar adsorption. Furthermore, the* R*_*L*_ value approaching 0 indicated that Cd adsorption on both biochars was an irreversible process (Fig. S3). Assessing the adsorption efficiency per unit area revealed that SBC had an adsorption efficiency of 16.75 mg m^−2^, surpassing ZBC, which had an adsorption efficiency of 3.54 mg m^−2^. By extending the comparison of Cd adsorption capacity to include a broader range of biochars from existing evidence (Table S2), it becomes apparent that SBC was more efficient in sorbing Cd, highlighting its potential for effective Cd remediation.

### The kinetics related in the Cd^2+^ adsorption

The adsorption capacity of the biochars for Cd^2+^ initially increased and then reached a plateau, indicating the attainment of equilibrium within 20 min (Fig. S4a). This rapid adsorption in the initial stage suggests that Cd^2+^ predominantly adsorbed onto the outer surfaces of the biochars. Over time, Cd^2+^ gradually diffused into the pores and reacted with internal active sites^41^. The adsorption kinetics of Cd^2+^ on the biochars followed the pseudo-second-order kinetic equation, as evidenced by high R^2^ values (> 0.91) and minimal differences between experimental and calculated equilibrium adsorption quantities (Table 2). These findings indicate that Cd adsorption by the biochars was a physiochemically controlled process involving electron sharing or exchange between the biochars and Cd^2+^^42,43^. Notably, the initial rate of Cd^2+^ adsorption (*v*_*0*_) was significantly higher for SBC compared to ZBC, indicating a stronger affinity of SBC for Cd^2+^. Furthermore, the results of Elovich model further revealed that SBC had much higher α values but lower β values than ZBC, suggesting that SBC possesses more active adsorption sites and a stronger electron-donating ability^44^.

**Table 2:** Fitting parameters for the kinetics of Cd adsorption on *S. europaea* biochar (SBC) and *Z. mays* biochar (ZBC) (biochar dosage 20 mg, initial concentrations of Cd^2+^ 30 mg L^−1^, contact time 0–120 min, pH 6.0, temperature 25 °C).

Kinetic model	Fitting parameter	SBC	ZBC
Pseudo—first order	*k*_1_ (min^−1^)	0.53	0.12
	*q*_*e*_ (mg g^−1^)	40.56	10.73
	R^2^	0.87	0.85
Pseudo—second order	*k*_2_ (min^−1^)	0.02	0.01
	*q*_*e*_ (mg g^−1^)	43.08	11.92
	R^2^	0.96	0.92
Elovich	α (mg g^*−*1^ h^*−*1^)	5070.52	5.70
	β (g mg^*−*1^)	0.26	0.45
	R^2^	0.96	0.96
	*v*_0_ (mg g^−1^ min^−1^)	40.83	1.99

The adsorption process of Cd^2+^ onto the biochars followed a three-phase mechanism, as observed from the intraparticle diffusion model (Fig. S4b, Table S3). These phases included boundary-layer diffusion (the initial diffusion of Cd^2+^ from the solution onto the biochar surface), intra-particle diffusion (the subsequent mass transfer of Cd^2+^ from the surface into the interior pores of the biochar), and dynamic equilibrium^45^. The findings confirmed the involvement of diffusion in Cd^2+^ adsorption onto the biochars, with pore filling occurring during the adsorption process^46^.

The rate constant (*k*_*p2*_) of SBC was higher than the values reported for other biochars, suggesting a more rapid adsorption process and a shorter adsorption time^47^. This difference could be attributed to the release of salt ions from the porous structure of SBC, thereby increasing the availability of adsorption sites within the pore structure. It is worth noting that the adsorption constant for each adsorption phase was not zero (Table S3), indicating that intraparticle diffusion of Cd^2+^ in the biochar was not the sole process at play. Other mechanisms, such as physical and chemical adsorption on the biochar surface, likely played significant roles, as supported by the pseudo-second-order model and and thermodynamic model. For instance, the values of ΔG^0^ in the present study fell within the ranges of − 20 to 0 kJ kJ mol^−1^ (Table S4), indicating a strong physical adsorption^48^.

### Processes of precipitation with minerals

**Figure 4:**
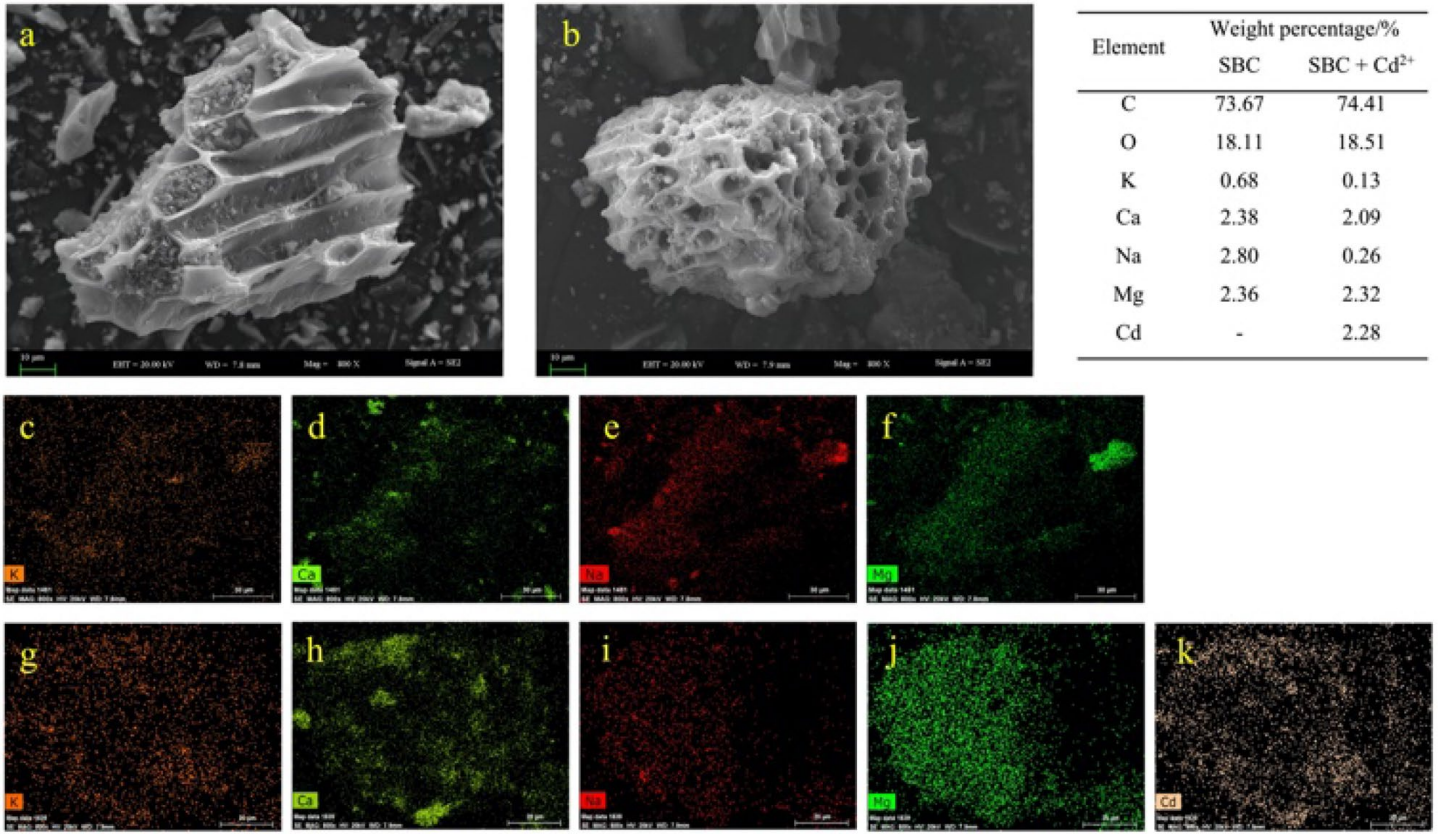
Scanning electron microscopy–energy dispersive X-ray energy spectroscopy (SEM–EDS) analysis of *S. europaea-* biochar (SBC). SEM images depicting SBC (**a**) and SBC loaded with Cd^2+^ (**b**). Elemental maps derived from EDX analysis showing the distribution of elements in SBC (K: **c**, Ca: **d**, Na: **e**, and Mg: **f**) and SBC loaded with Cd^2+^ (K: **g**, Ca: **h**, Na: **i**, Mg: **j**, and Cd: **k**). The table provides a comprehensive list of elemental contents.

In this study, the SEM images of the biochars before and after Cd^2+^ adsorption revealed the presence of particles or mineral crystals attached to the biochar surface.. The EDX elemental maps confirmed that these substances were Cd, indicating the formation of Cd-related compounds between Cd and the biochars (Figs. 4, S5).

**Figure 5:**
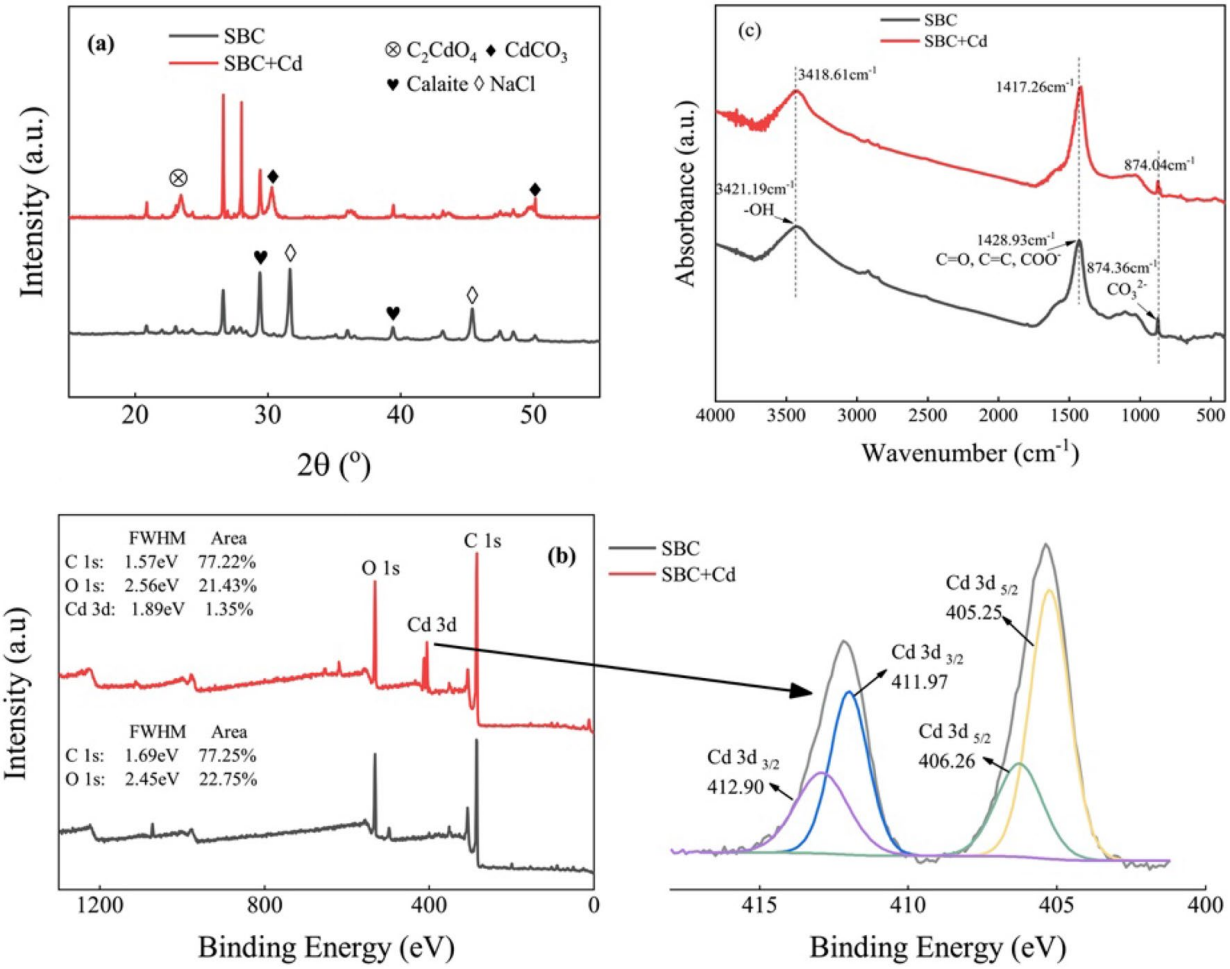
X-ray diffraction patterns (**a**), X-ray photoelectron spectroscopy patterns (**b**), and Fourier transform infrared spectra (**c**) of *S. europaea-*biochar (SBC). The *black* lines represent the original biochar, while the *red* lines depict the biochar loaded with Cd^2+^.

The XRD spectrum of SBC exhibited typical peaks associated with Cd minerals, including CdCO_3_ and C_2_CdO_4_ (Fig. 5a). In comparison, ZBC showed a peak of C_2_CdO_4_ (Fig. S6a). The precipitation of metals through adsorption can be attributed to the alkalinity of the biochars^49^. This mechanism was further confirmed by the variations in CO_3_^2−^ concentration in solution before and after Cd^2+^ adsorption (Fig. S7). Specifically, as the initial Cd^2+^ concentration increased, the release of CO_3_^2−^ into the solution was significantly reduced for SBC, indicating the involvement of CO_3_^2−^ in the Cd^2+^ adsorption process. In contrast, CO_3_^2−^ was not detected in the solution of ZBC. Our study demenstrated enhanced adsorption of Cd on SBC can be attributed to the precipitation of Cd with mineral CO_3_^2−^ phases of the biochar through complexation. This finding agrees with a previous study that found the formation of cadmium carbonate as a process for the adsorption of cadmium by biochar produced from giant miscanthus^50^. It should be noted that when the temperature exceeded 400 °C, the minerals (mainly CaCO_3_) present in the mixed biochar obtained by the co-pyrolysis of shrimp shell with corn straw decomposed into CO_2_^51^. However, in the case of SBC, a significant amount of CO_3_^2−^ was still observed even at 500 °C, indicating that carbonates are more conducive to retention in SBC.

In the XPS spectra, the presence of the Cd 3d peak was detected after Cd^2+^ adsorption on both biochars (Fig. 5b). The forms of Cd included Cd–O,–OCdOH, and CdCO_3_, with peaks in the range of 405–412 eV^52,53^. These findings suggest that the minerals present in SBC contributed to the adsorption of Cd^2+^, and Cd^2+^ precipitated in the form of inorganic carbonates or hydroxides.

### Processes of metal ion exchange

Some studies have suggested that the contribution of ion exchange to Cd^2+^ adsorption by biochar is negligible^9,54^. However, in our study, we observed a reduction in the weight percentages of K^+^, Ca^2+^, and Na^+^ on the biochar surfaces after Cd^2+^ adsorption (Fig. 4). Furthermore, the concentrations of K^+^, Ca^2+^, and Na^+^ in the solution increased following Cd adsorption (Fig. S8). By calculating the amount of Cd^2+^ sorbed through metal ion exchange, we found it to be 7.02 mg g^−1^ for SBC. These results indicate that metal ion exchange could be one of the mechanisms through which Cd^2+^ is sorbed onto SBC, in addition to other previously proposed mechanisms.

### Processes of surface complexation with oxygen-containing functional groups

The role of inner-sphere surface complexation with oxygen-containing functional groups in Cd^2+^ adsorption was assessed by analyzing the FTIR spectra of the biochars before and after Cd adsorption (Fig. 5c). The original SBC and ZBC biochars exhibited similar functional groups. The main functional groups, including hydroxyl groups (−OH) at 3400 cm^−1^ and carboxyl groups, ketones (C=O) or aromatic components (C=C), and carboxylate groups (COO−) in the range of 1380–1700 cm^−1^, were observed in both biochars. These hydroxyl and carboxyl groups can provide H ions, which are capable of undergoing ion exchange with metal ions^55,56^.

In our study, noticeable shifts in the bands at 3400 and 1400 cm^−1^ were observed subsequent to Cd^2+^ adsorption on the biochars, indicating the coordination between –COOH or –OH groups and Cd^2+^^17^. This coordination process often involves the release of H^+^ ions, resulting in a decline in solution pH. To confirm this, we measured the pH values of the solutions before and after Cd^2+^ adsorption by the demineralized SBCA and ZBCA biochars. The pH of the adsorption solutions decreased after Cd^2+^ adsorption on the demineralized biochars, providing further evidence for the complexation of Cd^2+^ with oxygen-containing functional groups on the biochar surfaces.

### Processes of Cd^2+^–π interactions

During pyrolysis process, aromatic structures are formed in biochar, with the cyclic aromatic π-system functions as a π-donor, donating electrons to Cd^2+^ during the adsorption process^57^. To investigate the nature of the adsorption mechanism, we analyzed the C 1s XPS spectra of the biochars before and after Cd^2+^ adsorption, as depicted in Fig. S9. Our analysis revealed the presence of unsaturated structures such as C=O, C–OH, C=C, and π–π* were present on the biochar surfaces. Importantly, the binding energies of aromatic carbonyl carbon (C=O) and hydroxyl carbon (C–OH) exhibited significant changes upon Cd adsorption onto the biochars, providing strong evidence for the involvement of Cd^2+^–π interactions as a contributing mechanism to the Cd^2+^ adsorption process of SBC.

### The contributions from different Cd^2+^ adsorption processes

**Figure 6:**
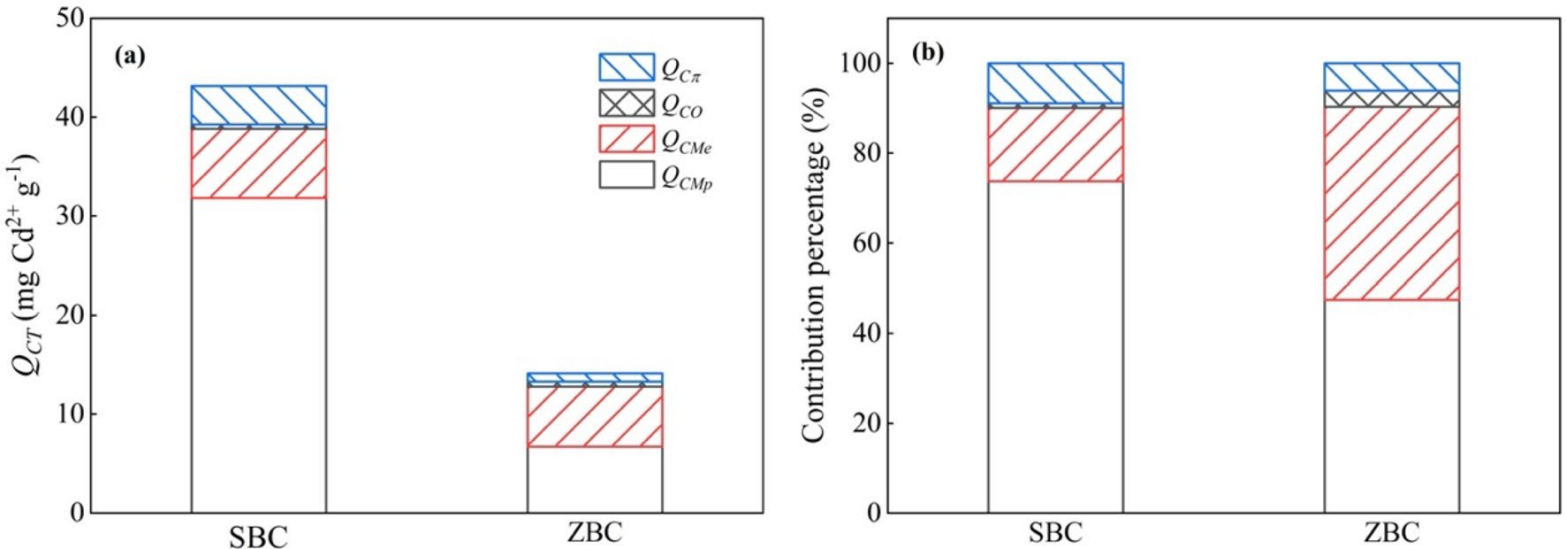
(**a**) Contributions of different mechanisms to Cd^2+^ sorption on *S. europaea* biochar (SBC) and *Z. mays* biochar (ZBC) and (**b**) the contribution percentage of the different mechanisms to overall Cd^2+^ adsorption: precipitation with minerals (*Q*_*CMp*_), metal ion exchange (*Q*_*CMe*_), functional group complexation (*Q*_*CO*_), and Cd^2+^–π interactions (*Q*_*Cπ*_).

The contribution of different mechanisms to the total adsorption of Cd^2+^ on both original and demineralised biochar was calculated and presented in Fig. 6. These mechanisms include precipitation with minerals (*Q*_*CMp*_), metal ion exchange (*Q*_*CMe*_), functional group complexation (*Q*_*CO*_), and Cd^2+^–π interactions (*Q*_*Cπ*_) to total Cd^2+^ adsorption (*Q*_*CT*_) on the biochars. The decreasing contributions for both biochars were ranked as follows: *Q*_*CMp*_ > *Q*_*CMe*_ > *Q*_*Cπ*_ > *Q*_*CO*_. Only 10% of total Cd^2+^ adsorption on the biochars was attributed to the organic components (*Q*_*CO*_/*Q*_*CT*_ + *Q*_*Cπ*_/*Q*_*CT*_), while the mineral components (*Q*_*CM*_) accounted for 90% of Cd^2+^ adsorption (*Q*_*CMp*_/*Q*_*CT*_ + *Q*_*CMe*_/*Q*_*CT*_). Liu et al.^58^ also considered that mineral precipitation was the main mechanism of Cd^2+^ adsorption by biochar. It is worth noting that the *Q*_*CMp*_ value of SBC was 31.81 mg Cd^2+^∙g^−1^, almost five times higher than that of ZBC. This difference can be attributed to the larger amount of CO_3_^2–^ released from SBC into solution before Cd^2+^ adsorption compared to ZBC. In this study, dominant mechanism of Cd^2+^ adsorption on the SBC was found to be the nteraction between minerals and Cd^2+^.

It is important to note that the physicochemical properties of biochar often undergo changes upon exposure to the environment, as evidenced by numerous studies^59,60^. For instance, as biochar ages, there is an observed increase in surface carboxyl and cation exchange capacity but a decrease in basicity^61^. These alterations may potentially impact its adsorption capacity for Cd. In this study, a short-term leaching test simulating aging revealed that the adsorption capacity of SBC at two concentrations (1% and 2%) did not exhibit a reduction (Fig. S10). However, the adsorption capacity of aged halophyte-biochar is highly expected to decrease beyond a certain threshold. Therefore, the impact of the long-term effects of halophyte-biochar on Cd adsorption certainly requires further evaluation.

## Conclusion

The study demonstrated the effective removal of Cd^2+^ from aqueous solution using biochar derived from *S. europaea*. The unique characteristics of this biochar, including its high pH, abundance of base cations, low surface negative charge, and high specific surface area, contributed to its effective adsorption of Cd^2+^. The study identified multiple mechanisms involved in Cd^2+^ adsorption, including precipitation, metal ion exchange, surface complexation, and Cd^2+^–π interactions. Among these mechanisms, the surface precipitation of CdCO_3_ was found to dominate the removal process, highlighting the importance of mineral interactions in Cd adsorption. Overall, this study provides insights into the diverse mechanisms involved in Cd^2+^ adsorption on *S. europaea*-derived biochar. The findings highlight the potential of utilizing biochars derived from euhalophyte plants as effective sorbents for Cd removal in contaminated water systems. Further research in this area can contribute to the development of sustainable and efficient approaches for water remediation and environmental protection.

### Supplementary Information

Supplementary Information.

## Data Availability

The datasets used and/or analysed during the current study available from the corresponding author on reasonable request.
