# Supplementary material for: Exploring adsorption capacity and mechanisms involved in cadmium removal from aqueous solutions by biochar derived from euhalophyte
Source: Sci Rep. 2024 Jan 3;14:450. doi: 10.1038/s41598-023-50525-2 (PMC10764732; doi:10.1038/s41598-023-50525-2)
Supplement: Supplementary file 1 — Supplementary Information. [file 41598_2023_50525_MOESM1_ESM.docx]

**Supplementary Material**

**Exploring adsorption capacity and mechanisms involved in cadmium removal from aqueous solutions by biochar derived from euhalophyte**

Shaoqing Ge ^a^, Shuai Zhao ^a,^*, Lei Wang ^a^, Zhenyong Zhao ^a^, Shoule Wang ^a,b^, Changyan Tian ^a,^*

a State Key Laboratory of Desert and Oasis Ecology, Xinjiang Institute of Ecology and Geography, Chinese Academy of Sciences, Urumqi 830011, China

b Shandong Institute of Pomology, Taian 271000, China

**This supplementary information contains:**

Number of Pages: 17

Number of Figures: 10

Number of Tables: 4

**1. Adsorption analysis equations**

The Langmuir model (Eq. 1) and Freundlich model (Eq. 2) were used to fit the isothermal adsorption curves of biochar:

$q_{e}=\frac{q_{m}bC_{e}}{1+bC_{e}}$ Eq. 1

$q_{e}=k_{f}C_{e}^{1/n}$ Eq. 2

where *q_e_* is the equilibrium adsorbed concentration of Cd(II) (mg⋅g^–1^), *q_m_* is the maximum adsorption capacity (mg⋅g^–1^), *C*_e_ is the equilibrium concentration (mg⋅L^–1^), *b* is the equilibrium Langmuir adsorption constant, *k_f_* is the Freundlich constant (mg^1–1/n^⋅g^–1^⋅L^1/n^), The 1/*n* is the Freundlich constant related to the surface site heterogeneity.

The Langmuir adsorption isotherm can also be defined as the dimensionless parameter *R*_L_:

$R_{L}=\frac{1}{1+bC_{0}}$ Eq. 3

where *R*_L_ is the separation factor (the affinity of the adsorbent for the adsorbate), and *C_0_* is the initial concentration of Cd(II) (mg⋅L^–1^). Normally, the adsorption process is irreversible for *R*_L_ ≅ 0, favorable for 0 < *R*_L_ < 1, linear for *R*_L_ = 1, and unfavorable for *R*_L_ > 1,

Pseudo-first-order (Eq. 4) and pseudo-second-order (Eq. 5) and the Elovich (Eq. 6) models were used to fit the adsorption kinetic curves of biochar:

$\ln\left( q_{e}-q_{t} \right)=\ln q_{e}-k_{1}t$ Eq. 4

$q_{t}=\frac{q_{e}^{2}k_{2}t}{1+q_{e}k_{2}t}$ Eq. 5

$q_{t}=\frac{1}{\beta}ln(\alpha\beta t+1)$ Eq. 6

where *q*_t_ is the adsorption capacity (mg⋅g^–1^) at time *t* (min), *q_e_* is the equilibrium adsorption capacity (mg⋅g^-1^), *k*_1_ is the pseudo-first-order rate constant (min^–1^), and *k*_2_ is the pseudo-second-order rate constant (g⋅mg^–1^⋅min^–1^), The parameters α (mg g^−1^ h^−1^) and β (g mg^−1^) are constants of Elovich.

The particle diffusion model (Eq. 7) was also used to describe the adsorption and diffusion processes of Cd on porous biochar:

*q*_t_ = *k_p_t*^0.5^ + *C* Eq. 7

where *k_p_* (mg g^−1^∙h^−0.5^) is the intraparticle diffusion rate constant, and *C* is a constant.

The thermodynamic parameters of adsorption process is obtained by the following equations:

$\Delta G^{0}=-RTlnK_{L}$ Eq. 8

$lnK_{L}=-\frac{\Delta H^{0}}{RT}+\frac{\Delta S^{0}}{R}$ Eq. 9

where ∆*H^0^* is the change of enthalpy (kJ∙mol^-1^), ∆*S^0^* is the change of entropy (J mol^-1^∙k^-1^), ∆*G^0^* (kJ∙mol^-1^) is the change of Gibbs free energy, *T* is the absolute temperature in Kelvin (K), *K_L_* (L∙mol^-1^) is the parameter of Langmuir isothermal model, and *R* is the gas constant (8.314 J mol^-1^ K^-1^).

References

1. Liu, L. & Fan, S. S. Removal of cadmium in aqueous solution using wheat straw biochar: effect of minerals and mechanism. *Environ. Sci. Pollut. R.* **25**, 8688–8700. https://doi.org/10.1007/s11356-017-1189-2 (2018).
2. Li, B. et al. Adsorption of Cd(II) from aqueous solutions by rape straw biochar derived from different modification processes. *Chemosphere.* **175**, 332–340. https://doi.org/10.1016/j.chemosphere.2017.02.061 (2017).
3. Han, L. F. et al. Removal of antimony (III) and cadmium (II) from aqueous solution using animal manure-derived hydrochars and pyrochars. *Bioresour. Technol.* **234**, 77–85. https://doi.org/10.1016/j.biortech.2017.02.130 (2017).
4. Bashir, S., Zhu, J., Fu, Q. L., Hu, H. Q. Comparing the adsorption mechanism of Cd by rice straw pristine and KOH-modified biochar. *Environ. Sci. Pollut. R.* **25**, 11875–11883. https://doi.org/10.1007/s11356-018-1292-z (2018).
5. Li, A. Y., Liu, L. H., Zhang, Y. T., Qiu, J. H. Synthesis and application of amine-functionalized MgFe2O4-biochar for the adsorption and immobilization of Cd(II) and Pb(II). *Chem. Eng. J*. **439**, 135785. https://doi.org/10.1016/j.cej.2022.135785 (2022).

| Samples | pH | Yield | Ash | Atomic ratio | | | Base cations (mol$\cdot$kg^−1^) | | | |
| --- | --- | --- | --- | --- | --- | --- | --- | --- | --- | --- |
|  |  | (%) | (%) | H/C | O/C | C/N | K^+^ | Ca^2+^ | Na^+^ | Mg^2+^ |
| SBC | 10.39 | 55.81 | 50.15 | 0.24 | 0.21 | 43.89 | 0.44 | 1.17 | 2.07 | 1.26 |
| ZBC | 9.09 | 35.25 | 24.67 | 0.26 | 0.31 | 41.19 | 0.75 | 0.34 | 0.11 | 0.33 |
| SBCA | 7.08 | 98.57 | - | 0.23 | 0.19 | 49.54 | - | - | - | - |
| ZBCA | 7.01 | 98.76 | - | 0.27 | 0.28 | 23.14 | - | - | - | - |

**Table S1** Physicochemical properties of *S. europaea* biochar (SBC) and *Z. mays* biochar (ZBC). H/C and O/C are the ratios among the atomic numbers of H, O, and C. C/N is the mass ratio of C to N.

| Adsorbent | Condition | Adsorption capacity (mg⋅g^−1^) | References |
| --- | --- | --- | --- |
| Wheat straw biochar | T=298K, pH=6 | 52.1 | Liu & Fan (*Environ. Sci. Pollut. R.* 2018, 25, 8688–8700) |
| Rape straw biochar | T=298K, pH=5.5 | 32.74 | Li et al. (*Chemosphere.* 2017, 175, 332–340) |
| swine solids biochar | T=298K, pH=4.5 | 79.89 | *Han et al.* (*Bioresour. Technol.* 2017, 234, 77–85) |
| rice straw biochar | T=298K, pH=5 | 12.17 | Bashir et al. (*Environ. Sci. Pollut. R.* 2018, 25, 11875–11883) |
| KOH-modified rice straw biochar | — | 41.9 |  |
| Rice husk biochar | T=298K, pH=5 | 9.98 | Li et al. (*Chem. Eng. J*. 2022, 439, 135785) |
| Carboxylated rice husk biochar | — | 31.23 |  |
| Zea mays-biochar | T=298K, pH=6 | 14.69 | This study |
| *Salicornia europaea* biochar | T=298K, pH=6 | 108.54 | This study |

**Table S2** Comparison of Cd adsorption effect on different biochar.

| Adsorption phases | Fitting parameter | SBC | ZBC |
| --- | --- | --- | --- |
| Boundary-layer diffusion | *K*_p1_ (mg g^-1^ h^-0.5^) | 21.86 | 3.63 |
|  | *C*_1_ | 0.93 | 0.15 |
|  | *R*^2^ | 0.98 | 0.98 |
| Intra-particle diffusion | *k*_p2_ (mg g^-1^ h^-0.5^) | 5.73 | 1.32 |
|  | *C*_2_ | 20.30 | 2.52 |
|  | *R*^2^ | 0.97 | 0.96 |
| Dynamic equilibrium | *k*_p3_ (mg g^-1^ h^-0.5^) | 0.24 | 0.22 |
|  | *C*_3_ | 41.01 | 9.80 |
|  | *R*^2^ | 0.80 | 0.72 |

**Table S3** Fitting parameters of the particle diffusion model for Cd adsorption on *S. europaea* biochar (SBC) and *Z. mays* biochar (ZBC). (biochar dosage 20 mg, initial concentrations of Cd^2+^ 30 mg L^-1^, contact time 0~120 min, pH 6.0, temperature 25 ^o^C).

| Adsorbent | T (K) | K_L_ (L⋅mol^−1^) | ΔG^0^ (kJ⋅mol^−1^) | ΔH^0^ (kJ⋅mol^−1^) | ΔS^0^ (kJ⋅mol^−1^) |
| --- | --- | --- | --- | --- | --- |
| SBC | 288.15 | 132.58 | -11.71 | -0.02 | 40.33 |
|  | 298.15 | 676.37 | -16.15 |  |  |
|  | 308.15 | 138.86 | -12.62 |  |  |
| ZBC | 288.15 | 113.10 | -11.32 | 0.09 | 55.16 |
|  | 298.15 | 139.98 | -12.24 |  |  |
|  | 308.15 | 265.40 | -14.29 |  |  |

**Table S4** Thermodynamic parameters for the adsorption of *S. europaea* biochar (SBC) and *Z. mays* biochar (ZBC). (biochar dosage 20 mg, initial concentrations of Cd^2+^ 30 mg L^-1^, contact time 0~120 min, pH 6.0).


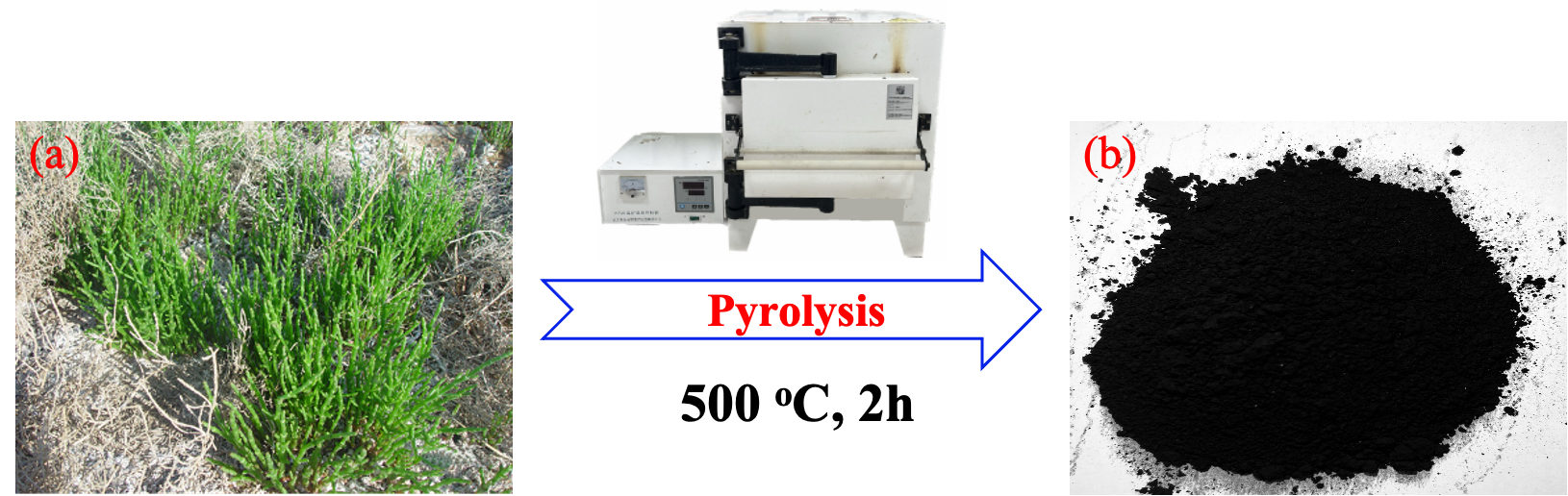


**Fig. S1** Photos for (a) raw *Salicornia europaea* plant and (b) biochar synthesized under pyrolysis condition of 500 °C and 2 h.


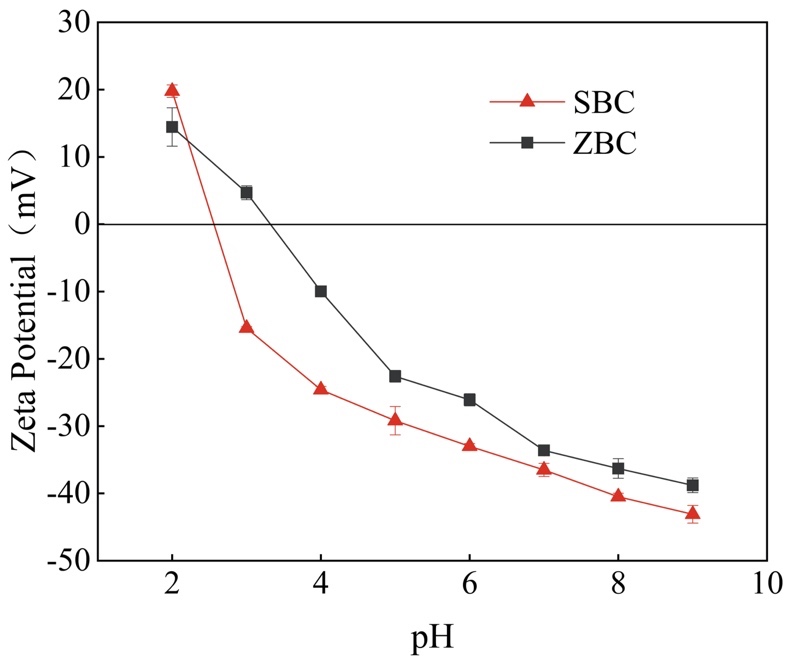


**Fig. S2** Zeta potentials of *S. europaea* biochar (SBC) (a) and *Z. mays* biochar (ZBC) as a function of solution pH.


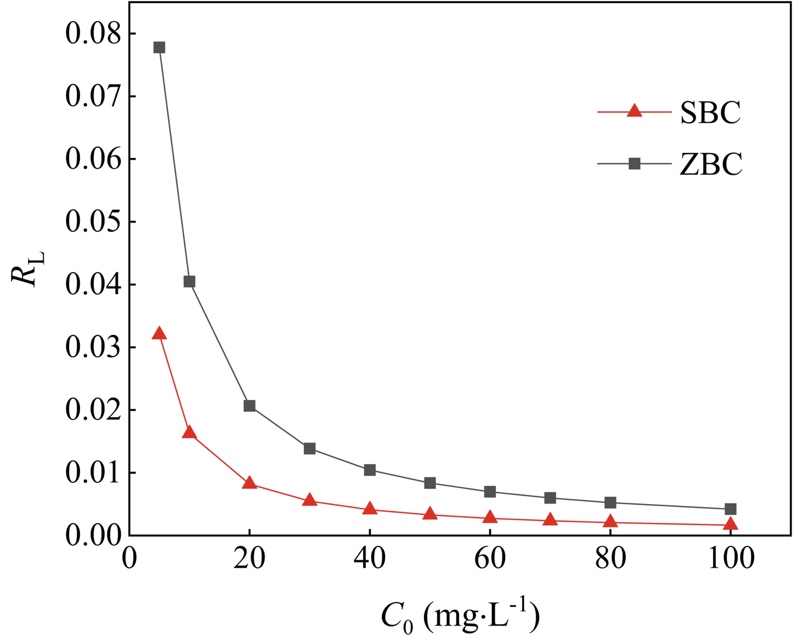


**Fig. S3** Relationship between the separation factor and the initial concentration of Cd^2+^.


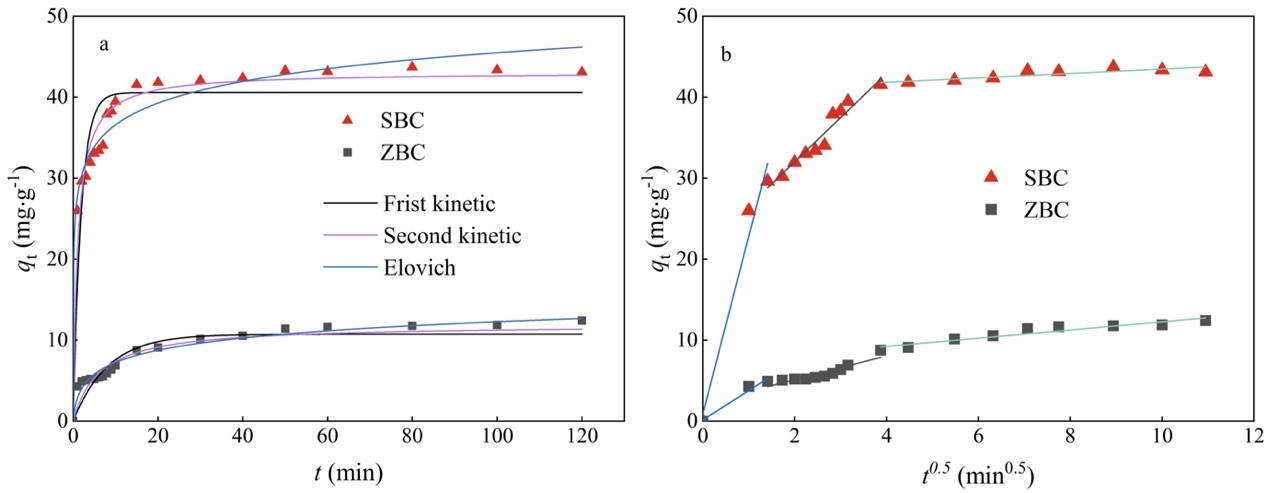


**Fig. S4** (a) Adsorption kinetics of Cd^2+^ by *S. europaea* biochar (SBC) (a) and *Z. mays* biochar (ZBC), (b) The intra-particle diffusion rate constants of Cd^2+^ adsorption by *S. europaea* biochar (SBC) (a) and *Z. mays* biochar (ZBC) (biochar dosage 20 mg, initial concentrations of Cd^2+^ 30 mg L^-1^, contact time 0~120 min, pH 6.0, temperature 25 ^o^C)..


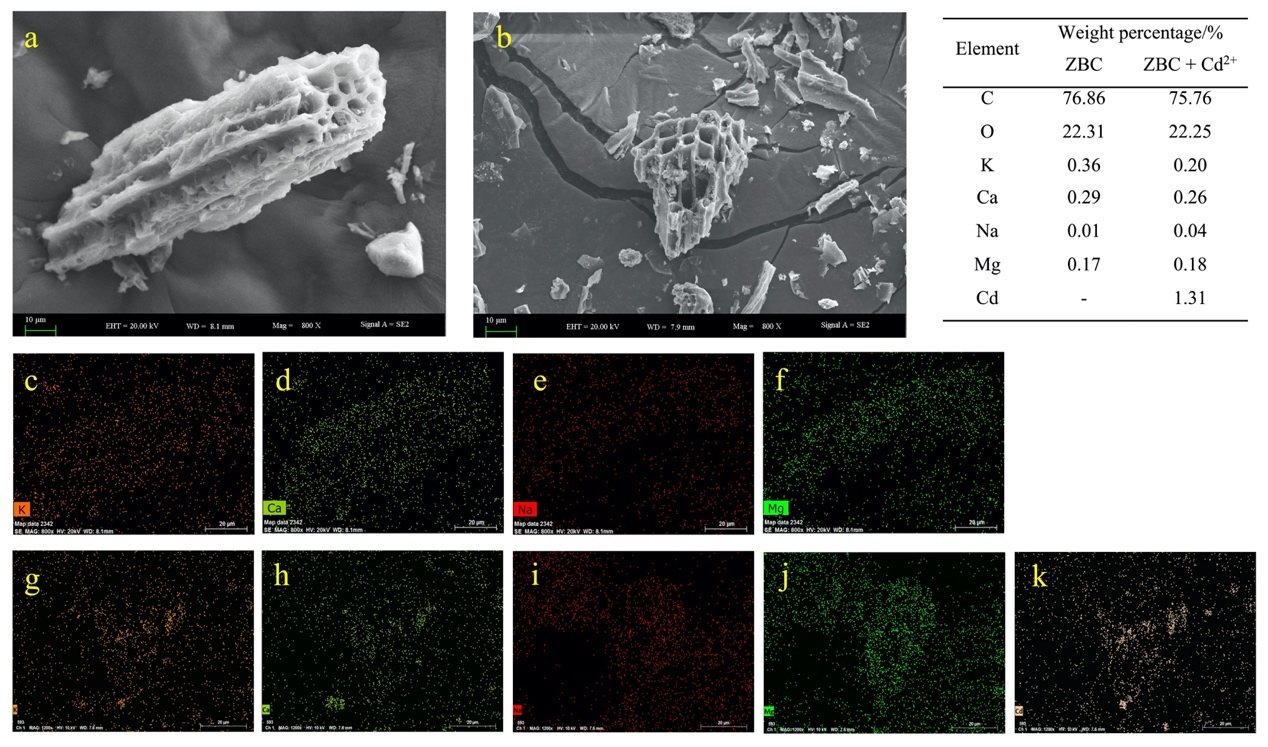


**Fig. S5** Scanning electron microscopy–energy dispersive X–ray spectroscopy (SEM-EDS) analysis of *Z. mays* biochar (ZBC). SEM images of ZBC (a) and ZBC loaded with Cd^2+^ (b). EDS elemental maps of ZBC (K: c, Ca: d, Na: e, and Mg: f) and ZBC loaded with Cd^2+^ (K: g, Ca: h, Na: i, Mg: j, and Cd: k). The table lists the elemental contents.


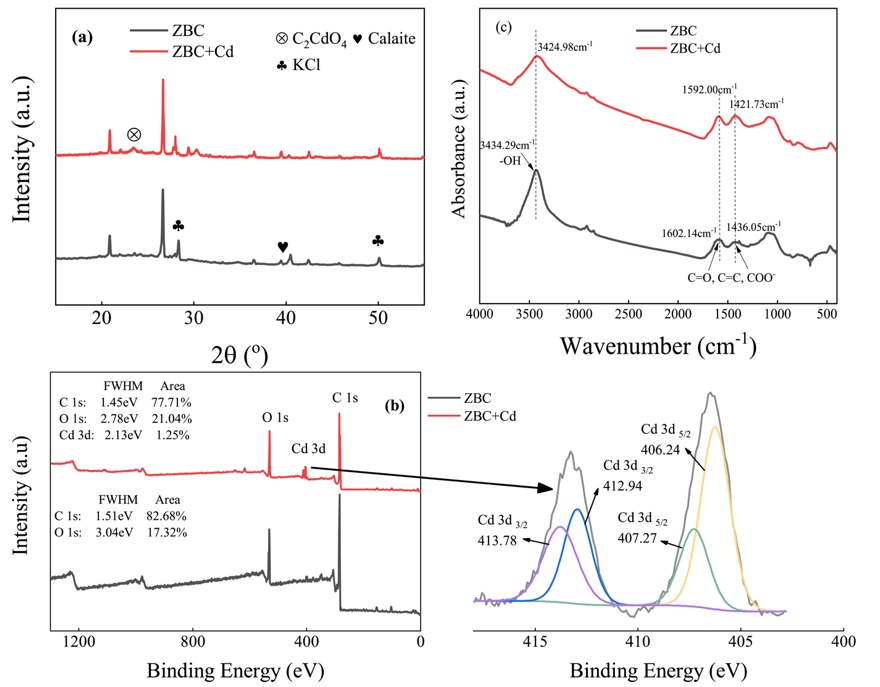


**Fig. S6** X-ray diffraction patterns (a), X-ray photoelectron spectroscopy patterns (b), and FTIR spectra (c) of *Z. mays* biochar (ZBC).


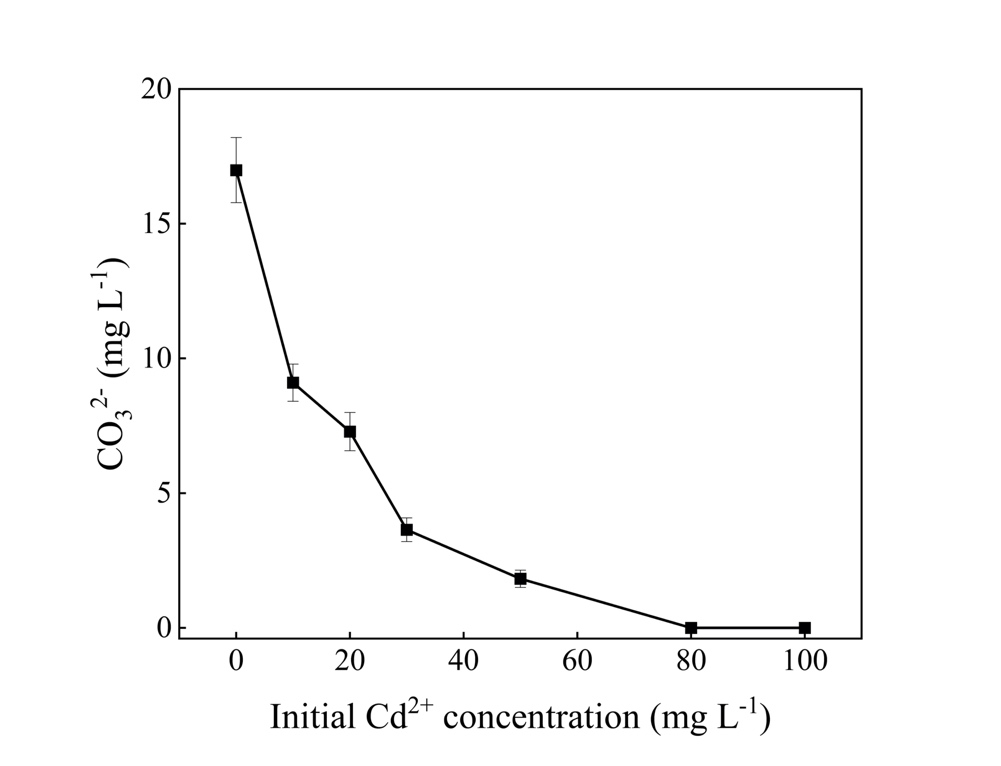


**Fig. S7** Variation in the solution CO_3_^2−^ concentration during Cd^2+^ adsorption on *S. europaea* biochar (SBC). Vertical bars represent standard deviation.


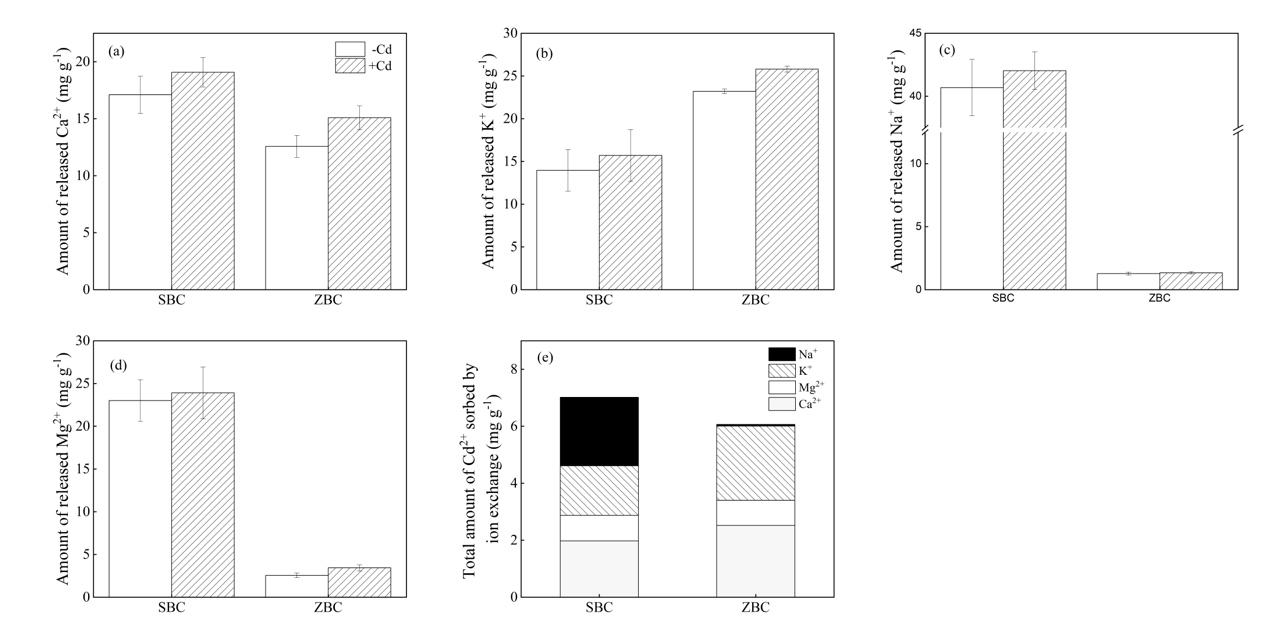


**Fig. S8** Amounts of Ca^2+^ (a), K^+^ (b), Na^+^ (c), and Mg^2+^ (d) released from biochar into solution before and after Cd^2+^ adsorption. The total amount of Cd^2+^ adsorbed by ion exchange (e). SBC: *S. europaea* biochar; ZBC: *Z. mays* biochar.


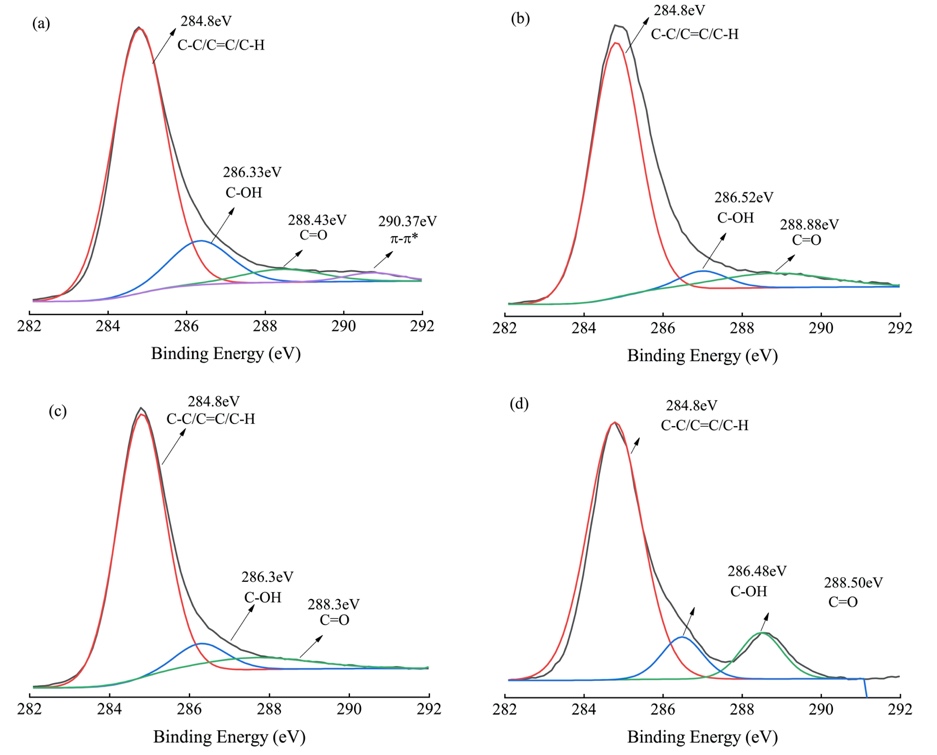


**Fig. S9** C 1s XPS spectra of the biochars before and after Cd adsorption. Before adsorption: *S. europaea* biochar (SBC) (a) and *Z. mays* biochar (ZBC) (c). After adsorption: *S. europaea* biochar (SBC) (b) and *Z. mays* biochar (ZBC) (d).


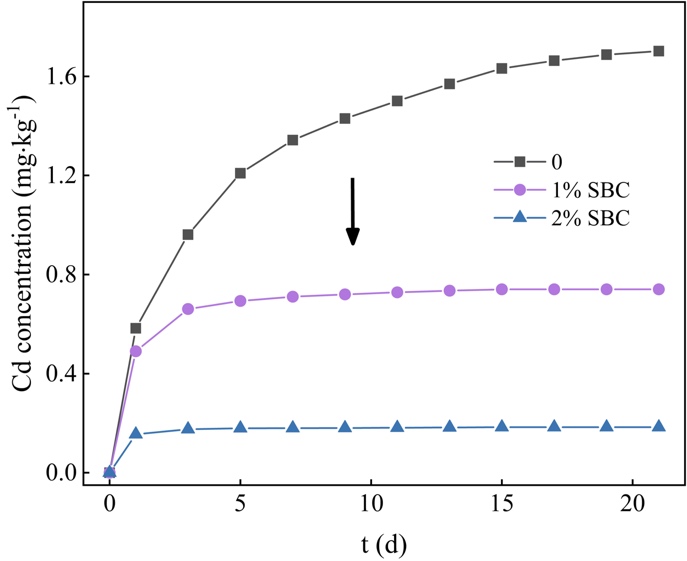


**Fig. S10** Cumulative amount of Cd leached from different treatment. 0 (10 mg∙kg^−1^ Cd, no biochar), 1% SBC (10 mg∙kg^−1^ Cd, 1% biochar) and 2% SBC (10 mg∙kg^−1^ Cd, 2% biochar). The arrow indicates stabilization of Cd by the biochar.
